# The Pathology of Intra-Uterine Death

**Published:** 1888-03

**Authors:** 


*The Pathology of Intra-uterine Death.* By W. O. PRIESTLEY, M.D., F.R.C.P. Pp. 192. London: J. & A. CHURCHILL. 1887.

As our readers are probably already familiar with the substance of this work, which is essentially the Lumleian Lectures for 1887, it is not necessary to do more than call attention to their publication in book form. They provide an admirable and clearly-written *resumé* of most that has, up to date, been written on the subject. There is very little that can be called original in the lectures, and we doubt whether anyone, either from exhaustiveness of presentment or from completeness of bibliography, would deem them sufficient basis from which to start original study. Probably they are intended mainly for the use of the much-petted, if somewhat mythical, "busy practitioner." To the favourable consideration of this gentleman we heartily commend the book. The few woodcuts we have seen before—scores of times.

